# Growth Hormone and Heart Failure: Implications for Patient Stratification, Prognosis, and Precision Medicine

**DOI:** 10.3390/diagnostics14242831

**Published:** 2024-12-16

**Authors:** Nikolaos Theodorakis, Magdalini Kreouzi, Christos Hitas, Dimitrios Anagnostou, Maria Nikolaou

**Affiliations:** 1 NT-CardioMetabolics, Clinic for Metabolism and Athletic Performance, 17564 Palaio Faliro, Greece; nikolaostheodorakis1997@yahoo.com; 2 School of Medicine, National and Kapodistrian University of Athens, 11527 Athens, Greece; 3 Department of Cardiology, Sismanogleio-Amalia Fleming General Hospital, 15127 Melissia, Greece; ch.chitas@flemig-hospital.gr (C.H.); jimdimitris100@gmail.com (D.A.); 4 Department of Internal Medicine, Sismanogleio-Amalia Fleming General Hospital, 15127 Melissia, Greece; kreouzi.m@live.unic.ac.cy

**Keywords:** growth hormone, insulin-like growth factor-I, heart failure, anabolic hormones, multiple hormonal deficiency syndrome, randomized controlled trials, precision medicine

## Abstract

Heart failure (HF) remains a major cause of morbidity and mortality worldwide. While standard treatments primarily target neurohormonal pathways, emerging evidence highlights the significant role of hormonal deficiencies, such as impaired growth hormone (GH) signaling, in HF progression and outcomes. GH is crucial for cardiovascular and skeletal muscle function, and its deficiency has been associated with worse prognosis. This review synthesizes recent findings from randomized controlled trials (RCTs) to explore how GH can contribute to personalized care and improve patient stratification in HF. A comprehensive literature review was conducted using PubMed up to 10 October 2024. Search terms included “growth hormone” combined with “heart failure”, “HFrEF”, “HFpEF”, and “HFmrEF.” Only placebo-controlled RCTs published in English and involving human subjects were considered. Data on study design, participant characteristics, GH dosing, and key clinical outcomes were systematically extracted and analyzed. Several trials demonstrated that GH therapy can transiently improve left ventricular ejection fraction (LVEF), exercise capacity, and reduce inflammatory markers. For example, one study has reported an increase in LVEF from 32 ± 3.8% to 43.8 ± 4.6% (*p* = 0.002), following three months of GH therapy in post-MI HF patients. However, benefits diminished after discontinuation. Additional studies have observed sustained improvements in peak oxygen consumption and LVEF over four years, with an additional trend towards hard endpoint improvement. Conversely, some studies showed no significant impact on cardiac function, highlighting heterogeneity in outcomes. As a result, GH therapy holds promise for improving cardiac and functional parameters in HF patients, but evidence remains mixed. Larger, long-term RCTs are needed to confirm its efficacy and safety. Precision medicine approaches and biomarker-driven strategies may optimize patient outcomes and guide clinical practice.

## 1. Introduction

**Figure 1:**
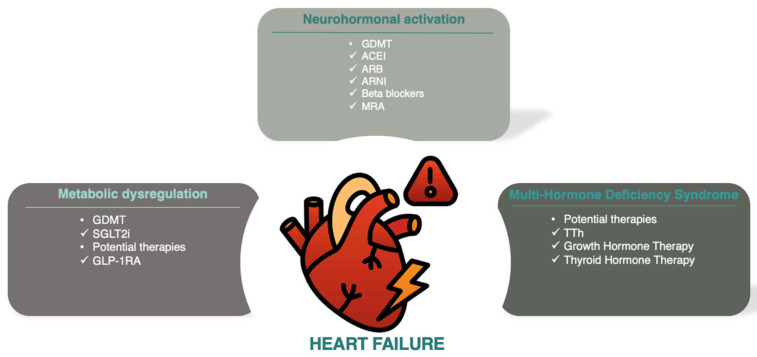
Schematic illustration of the main pathophysiologic pathways (neurohormonal, metabolic and MHDS) implicated in HF progression and prognosis. Abbreviations: ACEI (angiotensin-converting enzyme inhibitors); ARB (angiotensin receptor blockers); ARNI (angiotensin receptor-neprilysin inhibitors); GDMT (guideline-directed medical therapy); GLP-1RA (glucagon-like peptide-1 receptor antagonists); MRA (mineralocorticoid receptor antagonists); SGLT2i (sodium–glucose cotransporter-2 inhibitors), TTh (testosterone therapy).

Heart failure (HF) is a major cause of hospital admissions and ranks as the third leading cause of cardiovascular mortality, accounting for 25% of cardiovascular deaths globally [1]. Excessive activation of the sympathetic nervous system and the renin–angiotensin–aldosterone system drives HF with reduced ejection fraction, forming the basis for most guideline-directed medical therapies [2]. Beyond neurohormonal activation, deficits in anabolic hormones, known as multiple hormonal deficiency syndrome, have been associated with HF progression and worse outcomes [3]. A schematic illustration of the main pathophysiological pathways implicated in HF progression and prognosis is presented in Figure 1.

The TOSCA registry revealed that over 90% of HF patients had deficiencies in at least one anabolic hormone, such as testosterone, dehydroepiandrosterone sulfate, insulin-like growth factor-I (IGF-I), or triiodothyronine. More than two-thirds had multiple deficiencies, with nearly 50% experiencing IGF-I deficits, which were linked to a higher risk of hospitalization or death [3].

**Figure 2:**
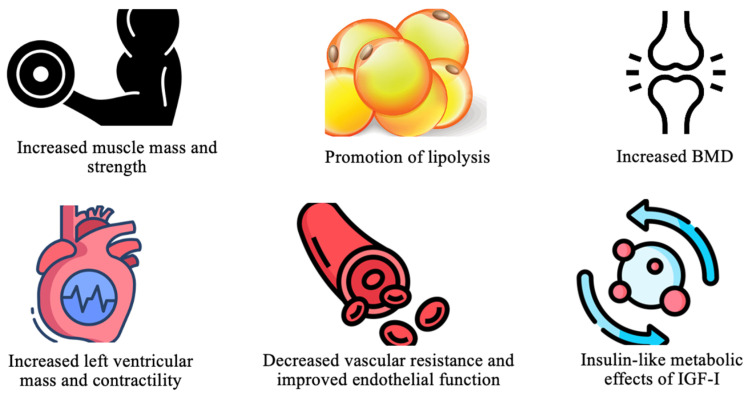
Key physiologic effects of GH. Abbreviations: BMD (bone mass density); IGF-I (insulin-like growth factor-1).

Growth hormone (GH) is essential for various physiological processes, primarily through the stimulation of hepatic IGF-I production. This action promotes protein synthesis, tissue repair, and cell growth [4]. In the cardiovascular system, GH enhances myocardial mass and contractility, lowers vascular resistance, and improves endothelial function. It also provides metabolic benefits, including increased muscle mass, bone density, and regulation of glucose metabolism through IGF-I [4]. These effects are depicted in Figure 2.

Recent meta-analyses of anabolic hormone therapies, such as thyroid hormone and testosterone, have shown promising results in chronic HF [5,6,7]. Regarding GH, despite its well-established physiological roles, the evidence regarding its diagnostic, prognostic, and therapeutic relevance in HF remains fragmented [8,9]. This review aims to provide an updated synthesis of current findings, emphasizing the potential of GH levels to improve patient stratification and prognosis and guide precision medicine strategies in HF.

## 2. Methods

A comprehensive literature search was conducted in PubMed up to October 10, 2024. The search strategy used terms such as “growth hormone” combined with “heart failure”, “HFrEF”, “HFpEF”, and “HFmrEF.” Only placebo-controlled, randomized controlled trials (RCTs) published in English and involving human subjects were included. Data extracted from each study included author, year, sample size, study duration, sex distribution, mean age (±SD), mean New York Heart Association (NYHA) class (±SD), mean left ventricular ejection fraction (LVEF) (±SD), mean baseline IGF-I levels (±SD), GH dose, and key clinical outcomes, as reported. Study selection and data extraction were performed independently by two reviewers, with disagreements resolved through consensus with a third reviewer.

## 3. GH for Advancing Precision Medicine Approaches in HF: Evidence from RCTs

From the search conducted, a total of 15 records were identified regarding RCTs investigating the use of GH therapy in HF [10,11,12,13,14,15,16,17,18,19,20,21,22,23,24].

### 3.1. Cardiac Remodeling and Function

GH exerts profound effects on myocardial structure and function, with its actions mediated directly and through its downstream effector, IGF-1. In HF, where ventricular remodeling and contractility are central to disease progression, GH has shown promise in promoting favorable structural and functional adaptations. A comprehensive, detailed analysis of GH’s effects on cardiac remodeling and function based on all published RCTs is provided below.

GH promotes myocardial hypertrophy through its anabolic properties, which include increased protein synthesis, enhanced myocardial cell growth, and reduced apoptosis. These changes are particularly important in the context of HF, where pathological remodeling is characterized by thinning walls, increased wall stress, and chamber dilatation. In Cittadini et al. (2009), GH therapy over six months resulted in a significant increase in LVPWd, rising from 7.8 ± 0.4 mm to 9.2 ± 0.5 mm (*p* < 0.01). This thickening reduced wall stress and contributed to improved systolic function. The hypertrophic effects were positively correlated with increases in serum IGF-1, indicating that the benefits of GH therapy extend through its endocrine pathways [10].

Perrot et al. (2001) found that GH therapy induced significant septal thickening, increasing left ventricular mass by 27% compared to placebo (*p* = 0.0001). The study identified IGF-1 elevation as a predictor of the magnitude of hypertrophy (r = 0.57, *p* < 0.001), suggesting a biomarker-guided approach to patient selection [11,12]. Napoli et al. (2002) documented a 15% reduction in LV end-diastolic volume and concomitant reductions in LV end-systolic volume (LVESV). These changes were indicative of a reversal of chamber dilatation and restoration of more efficient ventricular geometry [13]. In Cittadini et al. (2013), long-term GH therapy led to sustained decreases in LVESV by 20 ± 5 mL/m^2^, supporting its durable remodeling benefits in chronic HF patients [14].

One of the most critical outcomes of GH therapy is its impact on LVEF, a marker of systolic function and a predictor of prognosis in HF. Napoli et al. (2002) demonstrated a striking improvement in LVEF, which increased from 32 ± 3.2% to 43 ± 4.1% after three months of GH therapy (*p* = 0.005). The authors attributed this improvement to the combined effects of increased myocardial contractility and reduced wall stress [13]. Similarly, in Fazio et al. (2007), GH therapy increased LVEF from 32% to 43% (*p* < 0.005) over the same period, accompanied by significant gains in exercise capacity [15]. In the four-year follow-up study by Cittadini et al. (2013), GH therapy sustained a 10% improvement in LVEF, contrasting with a 2% decline in the control group (*p* < 0.001). These long-term effects were associated with reductions in adverse cardiac remodeling and hospitalizations [14]. Furthermore, in the study by Amirpour et al. (2021) LVEF was increased significantly following three months of GH therapy (32 ± 3.80% to 43.80 ± 4.60%, *p* = 0.002) [16].

While LVEF improvements were prominent in many studies, Isgaard et al. (1998) and Smit et al. (2001) reported no significant changes in LVEF, suggesting that patients with advanced HF or GH resistance may not derive similar benefits. In these cohorts, baseline IGF-1 levels and limited myocardial reserve may have constrained therapeutic effects [17,18]. GH therapy directly impacts left ventricular end-systolic wall stress (LVESWS), a critical determinant of myocardial oxygen demand and efficiency. By promoting myocardial hypertrophy and reducing chamber dimensions, GH therapy mitigates afterload and improves cardiac mechanics.

In the study by Adamopoulos et al. (2003), GH therapy significantly reduced LVESWS (*p* < 0.001), reflecting a reduction in myocardial strain during systole. These benefits were particularly pronounced in patients with severe LV dysfunction (LVEF <25%) [19]. In the study by Perrot et al. (2001), patients with the highest IGF-1 responses exhibited the greatest reductions in LVESWS, suggesting that biochemical responsiveness could guide therapy optimization [11,12]. In the study by Cittadini et al. (2013), long-term reductions in LVESWS were associated with sustained LVEF improvements, emphasizing the interplay between vascular function and myocardial mechanics [14].

The variability in response to GH therapy highlights the importance of patient selection. In the study of Karason et al. (2020), despite biochemical improvements (105% IGF-1 increase), no significant structural or functional changes were observed, suggesting that advanced ischemic HF may involve GH resistance [20]. In the study by Smit et al. (2001), the absence of LVEF or LVESWS improvements in their cohort further supports the need for tailored approaches, potentially involving higher doses or earlier intervention [18].

### 3.2. Exercise Capacity and Performance

Exercise capacity is a critical determinant of functional status and prognosis in HF. GH therapy has shown promising effects on cardiopulmonary performance, peak oxygen consumption (VO2max), and exercise duration in clinical studies.

VO2max is a gold-standard measure of aerobic capacity, reflecting the heart’s ability to deliver oxygenated blood to working muscles. GH therapy has been shown to improve VO2max significantly in HF patients. In the study by Napoli et al. (2002), a three-month study of HF patients (NYHA Class II-III), VO2max increased significantly from 20 ± 2 to 26 ± 2 mL/kg/min (*p* = 0.05) following GH therapy. These improvements were accompanied by enhanced endothelial function and reduced vascular resistance, suggesting better systemic oxygen delivery [13]. In the study by Fazio et al., VO2max improved from 19.8 ± 5.6 to 25.1 ± 5.6 mL/kg/min (*p* < 0.005) after three months of GH therapy. The authors attributed these gains to improved ventilatory efficiency and cardiac output [15]. The four-year follow-up study by Cittadini et al. (2013) demonstrated sustained improvements in VO2max, with an increase of 7.1 ± 0.7 mL/kg/min (*p* < 0.001) in GH-treated patients. This long-term benefit highlights GH’s role in maintaining functional capacity and delaying HF progression [14]. VO2max gains were linked to reduced left ventricular end-systolic wall stress (LVESWS) and enhanced contractility, as observed in Adamopoulos et al. (2003) [19].

Exercise duration reflects the combined effects of improved cardiac function, skeletal muscle oxygen delivery, and metabolic efficiency. GH therapy has consistently demonstrated the ability to prolong exercise duration in HF patients. In the study by Spallarossa et al. (1999), exercise duration increased significantly from 462 ± 121 s to 591 ± 105 s (*p* < 0.05) after six months of GH therapy. This 30% improvement underscores GH’s ability to enhance functional capacity. Notably, patients with severe ventricular dilatation (LV end-diastolic diameter >79 mm) did not achieve similar gains, suggesting the importance of patient selection [21]. In the study by Fazio et al. (2007), exercise duration improved by approximately 20% (*p* < 0.01) following GH therapy. The enhanced duration was attributed to better cardiopulmonary coupling and improved ventilatory efficiency [15].

Enhanced cardiac output and oxygen delivery to skeletal muscles were primary drivers of increased exercise duration. Napoli et al. (2002) highlighted improvements in endothelial function, as evidenced by increased nitric oxide bioavailability and reduced pulmonary vascular resistance during exercise (*p* < 0.03) [13].

Peak work capacity, a measure of the highest workload a patient can achieve during exercise, is closely linked to exercise duration and VO2max. Several studies reported significant improvements in peak work with GH therapy. In the study by Napoli et al. (2002), peak work improved significantly after three months of GH therapy, reflecting enhanced cardiac and muscular performance. This improvement was strongly associated with better ventilatory efficiency and reduced ventilatory equivalents for carbon dioxide [13]. In the study by Fazio et al. (2007), peak work capacity increased by 16 ± 3% (*p* < 0.05), aligning with reductions in left ventricular end-diastolic and end-systolic volumes. These changes highlight the direct impact of GH on cardiac mechanics and systemic performance [15].

GH therapy also positively influences ventilatory parameters, which are critical for assessing exercise performance in HF. In the study by Fazio et al. (2007), ventilatory efficiency, as measured by the VE/VCO2 slope, improved significantly following GH therapy (*p* < 0.005). Enhanced oxygen uptake kinetics contributed to a prolonged time-to-anaerobic threshold [15]. In the study by Adamopoulos et al. (2003), anaerobic threshold increased from 14.9 ± 4.8 to 20.0 ± 4.5 mL/kg/min (*p* < 0.005), indicating improved muscular oxygen utilization and delayed onset of metabolic acidosis [19].

Despite consistent findings of improved exercise capacity, not all patients benefit equally. In the study by Karason et al. (2020), despite biochemical improvements (105% IGF-1 increase), no significant changes in exercise performance were observed. The authors suggested that advanced ischemic HF may involve GH resistance or irreversible skeletal muscle pathology, limiting exercise benefits [20]. In the study by Isgaard et al. (1998), no improvements in exercise duration or peak work were observed in this study, emphasizing the need for tailored GH therapy based on baseline characteristics and disease severity [17].

### 3.3. Vascular Function and Hemodynamics

Vascular function and hemodynamic stability are critical determinants of HF progression and overall cardiovascular health. GH therapy, through its direct and IGF-1-mediated effects, has demonstrated significant potential to enhance vascular function, reduce systemic vascular resistance, and improve hemodynamic parameters in HF patients.

Endothelial dysfunction is a hallmark of HF, contributing to increased vascular stiffness, impaired vasodilation, and heightened cardiovascular risk. GH therapy has shown significant effects in improving endothelial function. In the study by Napoli et al. (2002), GH therapy significantly enhanced acetylcholine (ACh)-mediated endothelium-dependent vasodilation (*p* = 0.03). This was attributed to increased nitric oxide (NO) bioavailability, as evidenced by higher forearm nitrite and cGMP levels during ACh infusion (*p* = 0.05) [13]. Endothelium-independent vasodilation, assessed via sodium nitroprusside (SNP) response, also improved (*p* = 0.013), suggesting GH’s broader vascular benefits. In the study by Adamopoulos et al. (2003), GH treatment reduced levels of pro-inflammatory cytokines such as TNF-α and IL-6 (*p* < 0.05), contributing to endothelial repair and improved vascular function. The anti-inflammatory effects likely underpinned the observed improvements in vascular compliance. GH and IGF-1 enhance endothelial NO synthase (eNOS) activity, promoting NO production. This effect is critical for vasodilation, reduced vascular resistance, and improved oxygen delivery to peripheral tissues [19].

GH therapy reduces systemic vascular resistance (SVR) and pulmonary vascular resistance (PVR), directly influencing cardiac afterload and pulmonary hemodynamics. In the study by Napoli et al. (2002), PVR and SVR both decreased significantly after GH treatment, contributing to improved cardiac output and exercise capacity. These reductions were associated with increased NO production and enhanced vascular responsiveness [13]. Although not a primary endpoint in most studies, improvements in pulmonary pressures and vascular resistance were inferred from enhanced ventilatory efficiency and reduced VE/VCO2 slope in patients treated with GH. Fazio et al. (2007) reported these ventilatory improvements (*p* < 0.005), indirectly reflecting reduced pulmonary vascular burden [15].

GH therapy’s effects on systolic blood pressure (SBP) and diastolic blood pressure (DBP) reflect its influence on vascular tone and systemic resistance. In the study by Smit et al. (2001) [18], GH therapy reduced SBP by 15.8% (*p* < 0.05) in patients with advanced HF. The authors attributed this reduction to improved vascular compliance and decreased SVR. In the study by Napoli et al. (2002) [13], similar trends in SBP reductions were observed, further supporting the role of GH in afterload reduction. In the study by Smit et al. (2001), DBP was reduced by 8.2% (*p* < 0.05). These reductions in DBP align with GH’s systemic vasodilatory effects and enhanced vascular elasticity. In these studies, GH-induced blood pressure reductions did not lead to hypotensive episodes, highlighting its safety profile in hemodynamic modulation. In fact, GH therapy was associated with improved cardiac filling pressures and systemic perfusion [18].

While most studies observed significant vascular improvements, variability in response highlights the need for tailored therapy. In the study by Isgaard et al. (1998), despite biochemical evidence of GH activity, no significant vascular or hemodynamic improvements were observed, suggesting potential resistance mechanisms in advanced HF cohorts [17]. In the study by Karason et al. (2020), vascular benefits were limited in ischemic HF patients with chronic endothelial dysfunction, underscoring the importance of patient selection and timing of intervention [20].

### 3.4. Systemic Inflammation and Neurohormonal Activation

Systemic inflammation is a critical driver of HF progression. GH deficiency (GHD) exacerbates these processes by promoting pro-inflammatory states contributing to worsening cardiovascular outcomes. In the study by Adamopoulos et al. (2003), GHD was associated with significantly elevated tumor necrosis factor-alpha (TNF-α; 7.8 ± 1.1 pg/mL) and interleukin-6 (IL-6). GH therapy reduced TNF-α to 5.5 ± 0.9 pg/mL (*p* < 0.02), demonstrating the anti-inflammatory effects of GH [19]. In the study by Parissis et al. (2005), plasma IL-6 levels and soluble TNF receptors (sTNFRI and sTNFRII) were significantly elevated in HF patients with GHD. Furthermore, monocyte chemoattractant protein-1 (MCP-1), a potent chemokine implicated in vascular inflammation, was elevated in GHD patients and correlated with worsening HF. GH therapy significantly reduced MCP-1 levels (*p* < 0.05) [22].

In the study by Fazio et al. (2007), GH therapy reduced systemic markers of inflammation, including IL-6 and granulocyte–macrophage colony-stimulating factor (GM-CSF), contributing to improved endothelial and myocardial function (*p* < 0.05) [15]. In the study by Parissis et al. (2005), soluble Fas (sFas) and soluble Fas Ligand (sFasL), markers of apoptosis were significantly reduced following GH therapy, suggesting an anti-apoptotic role in mitigating HF pathophysiology (*p* < 0.05) [22]. In the study by Adamopoulos et al. (2003), anti-inflammatory/pro-inflammatory cytokine ratios, such as IL-10/TNF-α and TGF-β2/TNF-α, significantly improved with GH therapy (*p* < 0.05). Furthermore, soluble intercellular adhesion molecule-1 (sICAM-1) and vascular cell adhesion molecule-1 (sVCAM-1) levels were reduced after GH therapy (*p* < 0.05), indicating decreased endothelial activation and leukocyte recruitment [19]. These changes reflect a shift toward an anti-inflammatory state with GH therapy, which is critical for reversing HF progression.

### 3.5. Neurohormonal Activation

GHD exacerbates neurohormonal activation, as evidenced by elevated levels of stress-related hormones and cardiac biomarkers. GH therapy has shown significant benefits in mitigating this activation. In the study by Karason et al. (2020), GHD was associated with elevated N-terminal pro-brain natriuretic peptide (NT-proBNP) levels, a marker of cardiac stress. GH therapy reduced NT-proBNP levels by 48% (*p* < 0.001), reflecting improved myocardial strain and reduced neurohormonal activation [20]. In the study by Isgaard et al. (1998), elevated catecholamine levels were noted in GHD patients, reflecting sympathetic overactivation. GH therapy reduced these levels, suggesting partial normalization of neurohormonal balance [17]. In the study by Smit et al. (2001), RAAS activity, as evidenced by elevated plasma renin levels, was reduced with GH therapy, highlighting its systemic effects on neurohormonal pathways [18]. In the study by Cittadini et al. (2009), GH therapy reduced circulating levels of NT-proBNP, indicating a reduction in cardiac wall stress and improved ventricular performance (*p* < 0.01) [10].

IGF-1 plays a central role in mitigating inflammation and neurohormonal activation. In the study by Perrot et al. (2001), patients with higher baseline IGF-1 levels or significant IGF-1 increases (>80 pg/mL) demonstrated greater reductions in inflammatory markers and NT-proBNP levels, underscoring its importance as a biomarker for therapeutic response [11].

### 3.6. Safety of GH Therapy and Effect Adverse Events

The safety profile of GH therapy in HF patients, as demonstrated in the available RCTs, appears favorable compared to placebo. In the GH group, fewer deaths (9 vs. 13) and worsening HF events (16 vs. 31) were observed, indicating a potential benefit in reducing mortality and HF exacerbations. The occurrence of ventricular tachycardia (VT) was equal in both groups (two events each), suggesting no increased arrhythmic risk with GH. Furthermore, there were no cases of atrial fibrillation (AF) or bradyarrhythmias in the GH group, compared to four and one case(s), respectively, in the placebo group. When assessing composite adverse outcomes (death, worsening HF, or VT), the GH group exhibited substantially fewer events (27 vs. 46), further supporting its safety and potential efficacy [10,11,12,13,14,15,16,17,18,19,20,21,22,23,24].

### 3.7. Implications for Patient Stratification, Prognosis, and Precision Medicine

GH therapy in HF requires precise patient selection to maximize therapeutic benefits. Various studies provide insights into patient stratification based on clinical, biochemical, and functional parameters [10,11,12,13,14,15,16,17,18,19,20,21,22,23,24], listed as follows:

▪ Ischemic HF and Left Ventricular Function: Amirpour et al. (2021) highlighted that HF patients with a history of myocardial infarction (MI) in the left anterior descending artery and LVEF below 40% could benefit transiently from GH therapy [16]. Similarly, Karason et al. (2020) identified baseline IGF-1 and NT-proBNP levels as key markers for predicting treatment response in chronic ischemic HF [20].
▪ GHD: Cittadini et al. (2009, 2013) emphasized that patients with HF and documented GHD are ideal candidates, with baseline GH and IGF-1 levels, exercise capacity, and NT-proBNP levels serving as biomarkers [10,14].
▪ Dilated Cardiomyopathy (DCM): Fazio et al. (2007) and Parissis et al. (2005) noted that HF patients with DCM, moderate functional limitations (NYHA II-III), and severe LV dysfunction, especially those with elevated inflammatory markers, could be targeted for GH therapy. Baseline inflammatory profiles and cardiopulmonary performance metrics aid in identifying candidates [15,22].
▪ Inflammatory and Vascular Dysfunction: Adamopoulos et al. (2003) and Napoli et al. (2002) reported that patients with significant inflammatory activation (elevated TNF-α and IL-6) or vascular dysfunction (impaired endothelial reactivity and NO bioavailability) are more likely to benefit from the anti-inflammatory and endothelial effects of GH [13,19].
▪ Limited GH Responsiveness: Studies like van Thiel et al. (2004) and Acevedo et al. (2003) underscored the finding that HF patients with minimal IGF-1 increases and advanced ischemic HF or severe LV dysfunction (LVEF <30%) may not benefit significantly from GH therapy [23,24].
▪ Baseline Hormonal Profiles: Osterziel et al. (1998) and Perrot et al. (2001) highlighted the predictive value of low baseline IGF-1 levels and minimal cardiac remodeling in stratifying patients for GH therapy [11,12].

GH therapy’s impact on HF prognosis varies depending on patient selection and monitoring [10,11,12,13,14,15,16,17,18,19,20,21,22,23,24], indicated as follows:

▪ Transient and Sustained Benefits: Amirpour et al. (2021) observed transient LVEF improvements, emphasizing the need for continuous monitoring of GH levels and cardiac function [16]. By contrast, Cittadini et al. (2009, 2013) reported sustained improvements in LVEF and exercise capacity over four years in patients with GHD [10,14].
▪ Biochemical vs. Clinical Outcomes: Karason et al. (2020) noted that biochemical changes, such as elevated IGF-1, do not always correlate with structural or functional improvements, suggesting the limited prognostic value of biochemical markers alone [20].
▪ Exercise and Functional Metrics: Improvements in exercise tolerance and peak VO2, as observed by Fazio et al. (2007) and Spallarossa et al. (1999), are linked to better survival outcomes [15,21]. Monitoring these parameters could provide insights into treatment effectiveness.
▪ Inflammatory and Vascular Profiles: Reductions in inflammatory and apoptotic markers (Parissis et al., 2005; Adamopoulos et al., 2003) and enhancements in endothelial function (Napoli et al., 2002) suggest potential prognostic benefits in reducing cardiovascular risk and LV remodeling [13,19,22].
▪ GH Resistance: van Thiel et al. (2004) and Smit et al. (2001) emphasized GH resistance in advanced HF, underscoring the need for biomarkers to predict responsiveness and guide therapy [18,23].

The key findings and study designs are summarized in Table 1.

The implications for patient stratification, prognosis, and precision medicine regarding GH in HF are summarized in Table 2.

## 4. Gaps in Evidence and Future Research Directions

Despite promising therapeutic benefits observed in several studies, significant gaps remain in the evidence supporting GH therapy in HF management. Small sample sizes in many trials limit statistical power, potentially underestimating treatment effects and contributing to type II errors. While larger recent trials have shown more significant improvements in outcomes like LVEF and peak VO2, the short duration of GH treatment and limited follow-up periods hinder the assessment of long-term benefits and safety. Additionally, heterogeneity in HF etiology (e.g., ischemic vs. non-ischemic cardiomyopathy), severity, and baseline hormonal profiles complicates the interpretation of results and highlights the need for improved patient stratification. Variability in dosing regimens and administration protocols further complicates determining the optimal GH dose and duration to balance efficacy and safety.

GH resistance, particularly in advanced HF cases, presents another critical challenge, as it may diminish therapeutic effects. Mechanistic studies to elucidate GH resistance and identify biomarkers to predict responsiveness are essential. Personalized approaches using patient-specific characteristics, such as baseline IGF-I levels, inflammatory markers, and LV function, could optimize treatment strategies. Exploring GH therapy’s integration with standard HF treatments and evaluating combination approaches may yield synergistic effects and improve outcomes.

Future research should prioritize large-scale, multicenter RCTs with extended follow-up to provide robust evidence of GH therapy’s comprehensive efficacy and safety. Stratifying patients using biomarkers to identify those most likely to benefit from treatment is crucial. Additionally, precision medicine strategies that tailor interventions based on individual patient profiles represent a promising avenue for maximizing clinical benefits. By refining patient selection criteria, optimizing treatment protocols, and incorporating GH into existing HF therapies, future investigations may unlock its full therapeutic potential.

## 5. Conclusions

GH therapy shows promise as a treatment for HF, with evidence supporting its potential to improve cardiac function, exercise capacity, and metabolic parameters. However, limitations such as small sample sizes, short treatment durations, and variability in study designs underscore the need for further research. Addressing GH resistance, refining patient stratification strategies, and optimizing treatment protocols are critical. Large-scale trials integrating GH with standard therapies and applying precision medicine approaches hold promise for maximizing therapeutic benefits and establishing GH’s role in HF management.

## Figures and Tables

**Table 1:** Summary of RCTs on GH Therapy in HF.

Study	Design	Participants	Intervention	Key Outcomes
Amirpour et al. (2021) [16]	Double-blind RCT	16 HF patients post-MI, LVEF <40%	GH (5 IU every other day) for 3 months	LVEF: 32 ± 3.8% to 43.8 ± 4.6% (*p* = 0.002); ESD reduced: 39.43 ± 3.45 mm to 33 ± 3.16 mm (*p* = 0.03); No change in septum thickness or pulmonary pressure.
Karason et al. (2020) [20]	Double-blind RCT	37 DCM patients, EF <40%	GH (1.4 mg every other day) for 9 months	IGF-1 increased by 105% (*p* < 0.001); NT-proBNP reduced by 48% (*p* < 0.001); No significant changes in LV volumes, EF, or mass.
Cittadini et al. (2009, 2013) [10,14]	Single-blind RCT (2009) and long-term follow-up (2013)	56 HF patients with GHD, LVEF ~34%	GH replacement (0.012 mg/kg every other day) for 6 months (2009), 4 years follow-up (2013)	Peak VO2: 12.9 ± 0.9 to 14.5 ± 1.0 mL/kg/min (*p* < 0.01); EF increased from 34% to 36% (*p* < 0.01); Sustained EF rise of 10% over 4 years (*p* < 0.001).
Fazio et al. (2007) [15]	Double-blind RCT	22 HF patients, NYHA II-III	GH (4 IU every other day) for 3 months	IGF-I: 144 ± 35 to 293 ± 58 ng/mL (*p* < 0.005); Peak VO2: 19.8 ± 5.6 to 25.1 ± 5.6 mL/kg/min (*p* < 0.005); EF: 32% to 43% (*p* < 0.005).
Parissis et al. (2005) [22]	Randomized, crossover trial	12 DCM patients, NYHA III, LVEF ~24%	GH (4 IU every other day) for 3 months	TNF-α: 7.8 ± 1.1 to 5.5 ± 0.9 pg/mL (*p* < 0.02); peak VO2: 15.3 ± 0.7 to 17.1 ± 0.9 mL/kg/min (*p* < 0.01); LVESVI reduced: 128 ± 12 to 102 ± 12 mL/m^2^ (*p* < 0.001).
van Thiel et al. (2004) [23]	RCT	19 ischemic HF patients, LVEF <40%	GH (2 IU/day) for 6 months	IGF-I levels increased by 24% (*p* < 0.05); No change in LVEF, LV mass, EDV, or ESV.
Adamopoulos et al. (2003) [19]	Randomized, crossover	12 DCM patients, NYHA III, LVEF ~24%	GH (4 IU every other day) for 12 weeks	Endothelium-dependent vasodilation improved (*p* = 0.03); Peak VO2 increased from 20 ± 2 to 26 ± 2 mL/kg/min (*p* = 0.05); No change in LVEF.
Acevedo et al. (2003) [24]	Double-blind RCT	19 HF patients, LVEF <30%	GH (0.035 U/kg/day) for 8 weeks	LVESVI reduced (*p* < 0.001); Posterior wall thickness increased; Peak VO2 improved (*p* < 0.01); Inflammatory markers decreased significantly.
Napoli et al. (2002) [13]	Double-blind RCT	16 HF patients, NYHA II-III, LVEF <40%	GH (4 IU every other day) for 3 months	Increased LV mass by 27% (*p* < 0.0001); No change in LVEF or NYHA class; IGF-I increased by 77 ng/mL (*p* < 0.0001)
Smit et al. (2001) [18]	RCT	22 ischemic HF patients, LVEF <40%	GH (2.0 IU/day) for 6 months	Increased IGF-I, but no improvement in cardiac function or exercise capacity.
Osterziel et al. (1998)/Perrot et al. (2001) [11,12]	Double-blind RCT	50 DCM patients, LVEF <45%	GH (2 IU daily) for 12 weeks	No improvement in LVEF, exercise duration, or NYHA class; Increased IGF-I levels confirmed biological activity.
Spallarossa et al. (1999) [21]	Double-blind RCT	20 ischemic HF patients, NYHA II-III, LVEF <40%	GH (0.02 U/kg/day, titrated over 6 months)	Exercise duration increased from 462 ± 121 to 591 ± 105 s (*p* < 0.05); Improved well-being; IGF-I levels increased significantly; No change in LV function.
Isgaard et al. (1998) [17]	Double-blind RCT	22 DCM patients, LVEF <45%	GH (up to 4 IU daily) for 3 months	No significant change in LVMI, EDV, ESV, or systolic/diastolic function; IGF-I increased by 23.6% (*p* = 0.05).

Abbreviations. DCM (dilated cardiomyopathy); EDV (end-diastolic volume); EF (ejection fraction); ESD (end-systolic diameter); ESV (end-systolic volume); GH (growth hormone); GHD (growth hormone deficiency); HF (heart failure); IGF-I (insulin-like growth factor I); LV (left ventricle); LVESVI (left ventricular end-systolic volume index); LVMI (left ventricular mass index); MI (myocardial infarction); NT-proBNP (N-terminal pro B-type natriuretic peptide); NYHA (New York Heart Association); RCT (randomized controlled trial); TNF-α (tumor necrosis factor alpha); VO2 (oxygen consumption).

**Table 2:** Implications for patient stratification, prognosis, and precision medicine regarding GH in HF.

Study	Patient Stratification	Prognostic Implications	Presicion Medicine
Amirpour et al. (2021) [16]	Post-MI patients with LVEF <40%, MI location in LAD; GH responsiveness monitored via LVEF and end-systolic diameter changes.	Transient LVEF improvement; need for continuous monitoring post-GH discontinuation.	Tailored GH therapy based on MI location and LVEF; monitoring biomarkers for sustained benefits.
Karason et al. (2020) [20]	Chronic ischemic HF, LVEF <40%, IGF-1 and NT-proBNP levels as key biochemical markers for stratification.	Biochemical but not structural changes; unclear impact on cardiac outcomes; further monitoring needed.	Individualized treatment guided by IGF-1 and NT-proBNP; assessing long-term impact on HF management.
Cittadini et al. (2009, 2013) [10,14]	Patients with GH deficiency and HF; IGF-I levels, NT-proBNP, and exercise capacity used for stratification.	Sustained exercise and LVEF improvements; potential long-term benefits but require larger trials.	Precision approach targeting GHD in HF patients; personalized strategies based on biomarker profiles.
Fazio et al. (2007) [15]	Patients with DCM, NYHA class II-III; stratified by cardiopulmonary performance and echocardiographic parameters.	Cardiopulmonary and exercise capacity improvements linked to better survival outcomes.	Personalized strategies using exercise capacity and LV function to guide therapy.
Parissis et al. (2005) [22]	Patients with severe LV dysfunction, high inflammatory markers; cytokine profiles for identifying candidates.	Reductions in TNF-α and LV remodeling suggest improved prognosis in inflammatory HF cases.	Cytokine profiling for selecting patients likely to benefit from anti-inflammatory GH effects.
van Thiel et al. (2004) [23]	Advanced ischemic HF, LVEF <40%; GH responsiveness and resistance assessed, focusing on stage of HF.	No significant cardiac improvements; GH resistance considerations for advanced HF stages.	Focusing on patient-specific GH resistance mechanisms; potential use in early HF stages.
Acevedo et al. (2003) [24]	Severe LV dysfunction (LVEF <30%), low IGF-I responders; baseline IGF-I and neurohormonal markers considered.	Limited cardiac function benefits; possible focus on longer duration studies.	Long-term GH dosing trials and baseline biomarker assessments for patient-specific therapy.
Adamopoulos et al. (2003) [19]	DCM patients with significant inflammatory profiles; TNF-α and IL-6 as markers for therapy suitability.	Anti-inflammatory effects correlate with LV performance; inflammation reduction as a prognostic marker.	Using inflammatory markers to guide therapy and predict response; personalized anti-inflammatory approach.
Napoli et al. (2002) [13]	Chronic HF with vascular dysfunction; endothelial reactivity and NO bioavailability for patient selection.	Improved vascular function and exercise capacity; potential to reduce cardiovascular risk.	Endothelial function tests to select patients for therapy; addressing vascular dysfunction.
Smit et al. (2001) [18]	Ischemic HF, LVEF <40%; myocardial perfusion and IGF-I response as stratification criteria.	No functional improvements; need for further research on early intervention and tailored dosing.	Biomarker-based GH therapy for ischemic HF; evaluating myocardial perfusion.
Osterziel et al. (1998) and Perrot et al. (2001) [11,12]	DCM, LVEF <45%; IGF-I response patterns and LV mass increase as markers for patient selection.	High IGF-I responders show better outcomes; LV mass increase not always clinically beneficial.	Somatotrophic axis evaluation for tailored therapy; long-term monitoring of IGF-I response.
Spallarossa et al. (1999) [21]	Ischemic HF with moderate exercise intolerance; careful monitoring for arrhythmias and metabolic improvements.	Exercise tolerance improvement but arrhythmia risks; quality of life enhancements monitored.	Balancing exercise benefits with arrhythmia risks; personalized approaches for lipid profile improvements.
Isgaard et al. (1998) [17]	HF due to DCM, LVEF <45%; short-term GH therapy implications, with hyperglycemia risk monitoring.	Biological activity confirmed, but no clinical improvements; monitoring hyperglycemia and LV function.	Longer-term trials needed for personalized therapy; short-term safety monitoring, especially for hyperglycemia.

Abbreviations. HF (heart failure); DCM (dilated cardiomyopathy); GHD (growth hormone deficiency); GH (growth hormone); IGF-I (insulin-like growth factor-I); LAD (left anterior descending); LVEF (left ventricular ejection fraction); LV (left ventricular); NT-proBNP (N-terminal pro-brain natriuretic peptide); NYHA (New York Heart Association); NO (nitric oxide); TNF-α (tumor necrosis factor-alpha.
